# Diagnostic Stratification of Prostate Cancer Through Blood-Based Biochemical and Inflammatory Markers

**DOI:** 10.3390/diagnostics15111385

**Published:** 2025-05-30

**Authors:** Donatella Coradduzza, Leonardo Sibono, Alessandro Tedde, Sonia Marra, Maria Rosaria De Miglio, Angelo Zinellu, Serenella Medici, Arduino A. Mangoni, Massimiliano Grosso, Massimo Madonia, Ciriaco Carru

**Affiliations:** 1 Department of Biomedical Sciences, University of Sassari, 07100 Sassari, Italy; azinellu@uniss.it (A.Z.); carru@uniss.it (C.C.); 2 Department of Mechanical, Chemical, and Materials Engineering, University of Cagliari, 09123 Cagliari, Italy; leonardo.sibono@unica.it (L.S.); massimiliano.grosso@unica.it (M.G.); 3 Unit of Urology, University Hospital of Sassari (A.O.U. SS), 07100 Sassari, Italy; dott.alessandrotedde@gmail.com (A.T.); marra.sonia@gmail.com (S.M.); madonia@uniss.it (M.M.); 4 Department of Medicine, Surgery and Pharmacy, University of Sassari, 07100 Sassari, Italy; demiglio@uniss.it; 5 Department of Chemical, Physical, Mathematical and Natural Sciences, University of Sassari, 07100 Sassari, Italy; sere@uniss.it; 6 Department of Clinical Pharmacology, Flinders Medical Centre, Southern Adelaide Local Health Network, Adelaide 5042, Australia; arduino.mangoni@flinders.edu.au; 7 Discipline of Clinical Pharmacology, College of Medicine and Public Health, Flinders University, Adelaide 5042, Australia; 8 Unit of Oncology, University Hospital of Sassari (A.O.U. SS), 07100 Sassari, Italy

**Keywords:** biomarker discovery, prostate cancer, multivariate analysis, adaptive Box–Cox transformation, LASSO regression, probabilistic principal component analysis (PPCA), personalized medicine

## Abstract

**Background:** Prostate cancer (PCa) remains one of the most prevalent malignancies in men, with diagnostic challenges arising from the limited specificity of current biomarkers, like PSA. Improved stratification tools are essential to reduce overdiagnosis and guide personalized patient management. **Objective:** This study aimed to identify and validate clinical and hematological biomarkers capable of differentiating PCa from benign prostatic hyperplasia (BPH) and precancerous lesions (PL) using univariate and multivariate statistical methods. **Methods:** In a cohort of 514 patients with suspected PCa, we performed a univariate analysis (Kruskal–Wallis and ANOVA) with preprocessing via adaptive Box–Cox transformation and missing value imputation through probabilistic principal component analysis (PPCA). LASSO regression was used for variable selection and classification. An ROC curve analysis assessed diagnostic performance. **Results:** Five variables—age, PSA, Index %, hemoglobin (HGB), and the International Index of Erectile Function (IIEF)—were consistently significant across univariate and multivariate analyses. The LASSO regression achieved a classification accuracy of 70% and an AUC of 0.74. Biplot and post-hoc analyses confirmed partial separation between PCa and benign conditions. **Conclusions:** The integration of multivariate modeling with reconstructed clinical data enabled the identification of blood-based biomarkers with strong diagnostic potential. These routinely available, cost-effective indicators may support early PCa diagnosis and patient stratification, reducing unnecessary invasive procedures.

## 1. Introduction

Prostate cancer (PCa) is among the most prevalent cancers in men, accounting for approximately 15% of all cancers globally [1]. Its high incidence and associated mortality rates pose a significant public health challenge, particularly as the global population ages. By 2040, the global incidence of new PCa cases is projected to nearly triple, potentially reaching three million annually. The Lancet Commission has recently published projections indicating a significant increase in PCa cases from 1.4 million in 2020 to 2.9 million by 2040, with the greatest rise anticipated in low- and middle-income countries, where healthcare access and early detection programs remain limited [2].

This alarming trend underscores the growing burden of PCa, which not only affects patients and their families but also exerts significant pressure on healthcare systems worldwide.

Unlike some other cancers, this increase in PCa cases cannot be effectively mitigated through lifestyle changes or public health interventions, further emphasizing the urgent need for alternative strategies to address this issue [3,4].

Identifying effective biomarkers using advanced statistical methods is paramount among these strategies importance, as they hold promise for improving early diagnosis, risk stratification, and the development of personalized treatment strategies [5,6]. PCa predominantly affects older men, with over 60% of diagnoses occurring in individuals above the age of 65 [7]. The etiology of PCa is complex and multifactorial, involving a combination of genetic predispositions, such as BRCA1 and BRCA2 mutations, as well as environmental factors like diet, physical activity, and exposure to carcinogens [8,9,10]. Current diagnostic methods, including digital rectal examination (DRE) and prostate-specific antigen (PSA) tests, suffer from significant limitations in terms of specificity and sensitivity, resulting in the overdiagnosis of indolent cases and underdiagnosis of aggressive tumors [11,12]. While emerging imaging technologies, such as multiparametric magnetic resonance imaging (mpMRI), show potential to enhance diagnostic accuracy, their widespread adoption is constrained by the need for further validation and the high cost associated with implementation [13,14]. Consequently, treatment strategies for PCa have evolved to encompass a broad spectrum, ranging from active surveillance for low-risk tumors to radical prostatectomy, radiotherapy, and androgen deprivation therapy for high-risk cases [15,16] These approaches increasingly rely on personalized medicine, which takes into account factors such as individual metabolic pathways, pathological characteristics, patient preferences, comorbidities, and overall life expectancy [17]. Inflammation plays a critical role in cancer development and progression, including PCa [17,18,19,20]. This study aims to address these diagnostic gaps by investigating a panel of hematological and inflammatory biomarkers that could improve PCa stratification beyond PSA alone. The rationale for integrating these biomarkers stems from increasing evidence linking systemic inflammation and hematological changes to tumor progression and prognosis. While acute inflammation serves a protective role in fighting infections and promoting tissue repair, chronic inflammation is associated with DNA damage, immune evasion, and enhanced tumorigenesis [21,22,23]. Recent studies have highlighted the potential of inflammatory markers derived from routine blood tests, such as the neutrophil-to-lymphocyte ratio (NLR), platelet-to-lymphocyte ratio (PLR), and monocyte-to-lymphocyte ratio (MLR), as valuable prognostic tools in PCa [24]. Furthermore, hematological parameters such as hemoglobin (HGB), red cell distribution width (RDW), and hemoglobin distribution width (HDW) have also been shown to correlate with cancer outcomes, with anemia and elevated RDW frequently linked to poorer prognosis [25,26,27]. The role of eosinophils in PCa, through their dual involvement in anti-tumor immunity and tumor-promoting mechanisms, adds another layer of complexity and offers additional insights into disease progression [28,29,30].

Thus, the aim of this study was to integrate hematological and inflammatory biomarkers into a clinical model to predict the risk of positive prostate biopsy in patients suspected of prostate cancer. This approach could also assist in identifying clinically significant prostate cancer, thereby enhancing diagnostic precision and supporting personalized management strategies.

## 2. Materials and Methods

### 2.1. Study Design

This study was conducted on a cohort of 514 patients enrolled with clinical suspicion of prostate cancer (PCa) at the University Hospital of Sassari. Biological samples were collected from all the participants as part of a standardized diagnostic workflow. The screening protocol strictly adhered to the guidelines established by the Italian Association of Medical Oncology (AIOM), the Italian Society of Urology (SIU), and the Ministry of Health, with prostate biopsies serving as a pivotal component of the diagnostic process. Other procedures included comprehensive clinical evaluation, advanced imaging assessments, and blood-based biomarker analysis, leading to a diagnosis of benign prostatic hyperplasia (BPH), precancerous lesions (PL), or PCa.

This study received ethical approval from the Independent Ethics Committee of the Azienda Ospedaliero Universitaria di Cagliari (Approval Code: Prot. PG/2022/4985, Approval Date: 30 March 2022), in compliance with the Declaration of Helsinki and international guidelines for good clinical practice. Ethical considerations were rigorously upheld to ensure participant safety, confidentiality, and informed consent. A flow diagram detailing the patient journey, from initial presentation to final diagnosis, is provided in the graphical abstract. The eligibility criteria included male patients over the age of 50 presenting with clinical or biochemical indications of PCa, such as elevated serum PSA concentrations, abnormal findings on DRE, or radiological abnormalities. Patients with a prior history of PCa, ongoing or completed treatment for any malignancy, severe comorbidities contraindicating biopsy, or incomplete clinical data were excluded from this study.

#### Diagnostic Workflow and Histological Confirmation

All the patients included in this study (n = 514) were enrolled based on clinical suspicion of prostate cancer (PCa), following international guidelines. The diagnostic protocol adhered to a standardized, multi-step approach, consistent with recommendations from the Italian Association of Medical Oncology (AIOM) and the Italian Society of Urology (SIU), and was applied uniformly to all the participants. The diagnostic process included the following:

Initial clinical evaluation, including digital rectal examination (DRE);
Serum prostate-specific antigen (PSA) measurement (performed in all patients);
Multiparametric magnetic resonance imaging (mpMRI) in 378 out of 514 patients (73.5%), evaluated according to PI-RADS v2.0 criteria. A PI-RADS score ≥ 3 prompted further evaluation;
Transrectal ultrasound (TRUS)-guided prostate biopsy, performed in all patients with a minimum of 12 cores sampled;
Histopathological evaluation of biopsy specimens, based on Gleason scoring and assignment to International Society of Urological Pathology (ISUP) Grade Groups;
In selected cases, radical prostatectomy was performed to confirm ISUP grading and assess pathological staging (pT, pN);
Radiological staging (e.g., CT, bone scintigraphy, or PSMA-PET) was performed in patients with PSA > 20 ng/mL or ISUP grade ≥ 3 to evaluate potential metastatic disease.

The diagnostic outcomes were histologically confirmed PCa, benign prostatic hyperplasia (BPH), or precancerous lesions (PL). The endpoint for classification was defined by the result of the biopsy. For PCa patients, ISUP Grade Groups were recorded for stratification purposes. When available, radical prostatectomy findings were used to validate initial biopsy-based diagnoses. The primary focus of the present study was the diagnostic evaluation before the initiation of any treatment.

### 2.2. Clinical and Laboratory Parameters

The following clinical, hematological, and inflammatory parameters were assessed for association with positive prostate biopsy outcomes: age, serum total PSA, hemoglobin (HGB), red blood cell count (RBC), white blood cell count (WBC), neutrophil count, neutrophil-to-lymphocyte ratio (NLR), systemic inflammatory response index (SIRI), hemoglobin distribution width (HDW), and erectile function score (International Index of Erectile Function, IIEF-5). Laboratory parameters were measured using standard automated hematology analyzers following international protocols.

### 2.3. Statistical Analysis

The data analysis was conducted using both univariate and multivariate approaches to ensure robust identification of significant variables. Statistical analyses were performed using GraphPad Prism (version 10.0.2, GraphPad Software, San Diego, CA, USA) and R software (version 4.2.3, R Foundation for Statistical Computing, Vienna, Austria).The initial examination of the dataset revealed substantial deviations from Gaussian distributions, leading to highly skewed data. To address this, two univariate methods were applied. First, the Kruskal–Wallis (KW) test, a non-parametric approach, was used to identify variables with significant differences in median values across groups. Second, the adaptive Box–Cox (ABC) transformation was employed to reshape variables to approximate Gaussian distributions, enabling the application of parametric tests such as ANOVA [31,32,33,34]. The results of the Lilliefors normality test applied to the data before and after the ABC transformation are presented in Supplementary Materials, Section S1.

The dataset contained approximately 7% missing values. To tackle this issue, we employed a probabilistic principal component analysis (PPCA). This approach models the data within a Gaussian latent variable framework, extending traditional principal component analysis to handle noise and missing data through maximum likelihood estimation. It has been shown that PPCA effectively estimates missing data, even in validation scenarios [35]. This method was applied to the ABC-transformed data, as PPCA performs well under the normality assumption.

The number of probabilistic principal components was 14, as they allowed for achieving 95% of the cumulative explained variance. Before performing PPCA, autoscaling was applied as a preprocessing step due to the diverse orders of magnitude across the different ABC-transformed variables. After the missing data imputation, the dataset was mapped back to its original space. Imputing missing values is crucial for enhancing the effectiveness of the ANOVA test, as it enables the inclusion of more data points. The data resulting from this combined operation will be hereafter referred to as ABC-PPCA reconstructed data.

On the reconstructed dataset, ANOVA was conducted to evaluate group differences, followed by Tukey–Kramer post-hoc tests to identify significant pairwise comparisons. The significance level for all the hypothesis tests was set at *p* < 0.05. In order to avoid potential false discovery errors from multiple comparisons, the Benjamini–Hochberg correction was applied with a false discovery rate (FDR) threshold of 5% [36].

From a multivariate perspective, the least absolute shrinkage and selection operator (LASSO) regression was employed to perform variable selection and evaluate their discriminatory power [37]. LASSO regression allows for both variable selection and fitting by identifying the coefficient values of a multiple linear regression such that an L1-penalized least squares objective function is minimized [38]. The solution of the problem generates a quasi-sparse coefficients vector. However, only BPH and PCa classes were considered in this case, since no clear separation was observed when the PL group was contemplated in the dataset, as reported later in the results section. In this regression analysis, membership in the BPH and PCa groups was represented by the numbers 1 and 2, respectively. For the test set, if the predicted response exceeded 1.54, the model assigned the sample to the PC class; otherwise, it was attributed to the BPH class. The optimal value of the penalization parameter was determined through Monte Carlo cross-validation with 5000 iterations (for each iteration, 30% of samples were used as a test set). Therefore, the optimal combination of cutoff and penalization parameter values was selected based on the highest achieved accuracy. Standard normal variate (SNV) preprocessing was applied before performing the regression to analyze the absolute values of the regression coefficients and compare their relative importance in discriminating the different classes. The features commonly selected by the three methods (Kruskal–Wallis, Box–Cox ANOVA, and LASSO) were recognized as significant for this study.

## 3. Results

### 3.1. Univariate Analysis

Table 1 summarizes the significant clinical variables identified through univariate analysis, presenting the corresponding parameter values following ABC transformation. The effect size for the one-way ANOVA, as measured by Cohen’s *f*, is reported in the Supplementary Material, Section S2.

The Kruskal–Wallis test identified statistically significant differences across groups in 11 variables: age, PSA, Index %, red blood cells (RBC), HGB, neutrophils, LMR, NLR, systemic inflammatory response index (SIRI), International Index of Erectile Function (IIEF), and transrectal ultrasound scan (TRUSS). The preprocessing step confirmed deviations from normality in these variables, reinforcing the necessity of transformation before further comparisons.

The further assessment of the ABC-PPCA reconstructed data confirmed statistical significance for 10 variables: age, PSA, Index %, HGB, RBC, HDW, neutrophils, NLR, SIRI, and IIEF [39]. While LMR and TRUSS were initially identified as significant, their effect was not confirmed in subsequent analyses. Conversely, HDW, which was not initially highlighted, emerged as significant in this adjusted dataset, suggesting borderline significance for certain variables. To minimize the risk of false positives, FDR correction was applied. This adjustment confirmed age, PSA, Index %, HGB, and IIEF as the most robust biomarkers, consistently distinguishing between patient groups. These findings reinforce their potential relevance in clinical stratification and disease characterization. A sensitivity analysis was performed to evaluate the normality of the distributions across different λ values, using the *p*-values from the Lilliefors test. This analysis was conducted for each variable and within each class. The detailed results are available in the Supplementary Material, Section S3.

The clinical, hematological, and inflammatory parameters evaluated in patients with benign prostatic hyperplasia (BPH), precancerous lesions (PLs), and prostate cancer (PCa) are summarized in Table 2. Mean values and standard deviations (SDs) for each variable are reported. Statistical comparisons among groups were performed using a one-way ANOVA followed by post hoc Tukey tests. The same table also reports the class for which the variable is upregulated. As can be observed, most markers are upregulated in the PCa class. In contrast, higher values of other variables, such as IIEF, are reasonably associated with patients in the PBH class.

In order to assess the potential bias introduced by data imputation, we calculated the R2 coefficient on the previously known (non-missing) data prior to probabilistic PCA, obtaining a value of 0.99992. Additionally, we generated violin plots of the residuals for all the investigated variables, provided in the Supplementary Materials. These results confirm the reliability of the imputation process, indicating that no artificial significance was introduced. A detailed ISUP-based subgroup analysis is provided in Supplementary Section S4, including the distribution of patients across ISUP grades and associated variations in clinical and inflammatory biomarkers (Table S4).

### 3.2. Distribution of ISUP Grades in Confirmed PCa Patients

Among the 275 patients with histologically confirmed prostate cancer (PCa), the distribution across ISUP Grade Groups is reported in Supplementary Table S3. Briefly, 42.2% of patients were classified as ISUP Grade 1, 13.1% as Grade 2, 12.4% as Grade 3, 20.0% as Grade 4, and 12.4% as Grade 5. This distribution indicates a predominance of lower-grade disease (Grades 1–2), with higher-grade cancers (Grades 4–5) accounting for a significant proportion of aggressive cases.

Radical prostatectomy was performed in 63 out of the 275 PCa patients (22.9%), allowing for the pathological confirmation of ISUP grading and tumor staging (pT and pN). Radiological staging was performed in 64 patients with PSA > 20 ng/mL or ISUP Grade ≥ 3, identifying metastatic disease in 19 cases (7.0%).

Further stratification of clinical and hematological parameters across ISUP grades, including PSA, hemoglobin, white blood cell count, neutrophil-to-lymphocyte ratio (NLR), and systemic inflammatory response index (SIRI), is detailed in Supplementary Table S4.

### 3.3. Post-Hoc Analysis

Pairwise comparisons using a post-hoc analysis identified significant differences primarily between the BPH and PCa groups, while no significant variations were observed between BPH and PL. However, comparisons between PL and PCa revealed significant differences for PSA, Index %, HGB, HDW, and IIEF, indicating their potential role in distinguishing these two conditions (Table 3).

The RBC parameter exhibited a borderline *p*-value slightly above 0.05, likely due to its marginal significance in the initial analysis. This result suggests a reduction in statistical power when transitioning from an overall comparison to pairwise testing. These findings highlight the importance of appropriate data preprocessing and statistical correction methods to ensure robust and reliable conclusions.

### 3.4. Multivariate Analysis

**Figure 1:**
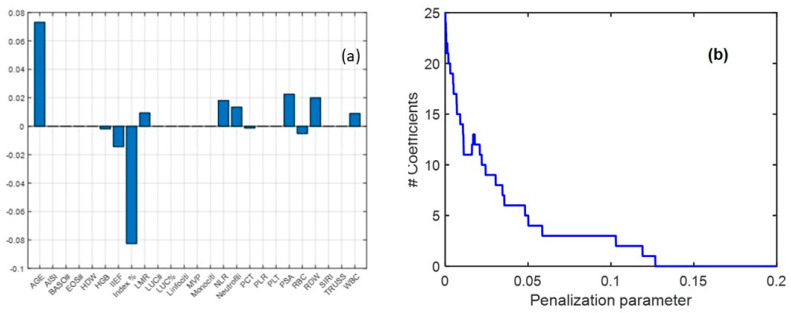
Coefficient values obtained from the LASSO regression (**a**) and sensitivity analysis for LASSO coefficients by varying the penalization parameter (**b**). The plot illustrates the relationship between the penalization parameter (λ) and the number of nonzero coefficients retained in the model. As λ increases, the number of selected variables decreases, demonstrating the sparsity-inducing property of LASSO.

Because the most significant differences in the univariate analysis were observed between the BPH and PCa groups, the PL class was excluded from the subsequent multivariate analysis. In this analysis, LASSO regression was applied, serving both as a variable selection technique and a classification tool. The analysis identified 12 nonzero regression coefficients, confirming their contribution to classification. Age, Index %, PSA, NLR, and neutrophils emerged as the most influential biomarkers, consistently with univariate findings. Among these, age and Index% exhibited the highest regression coefficients, reinforcing their diagnostic relevance. Additionally, PSA, RBC, NLR, neutrophils, and IIEF, which were identified as significant in prior analyses, also demonstrated strong discriminatory power in the LASSO model (Figure 1a). These findings support the clinical utility of a selected biomarker panel for distinguishing between BPH and PCa, highlighting their potential role in refining diagnostic strategies and improving patient stratification.

**Figure 2:**
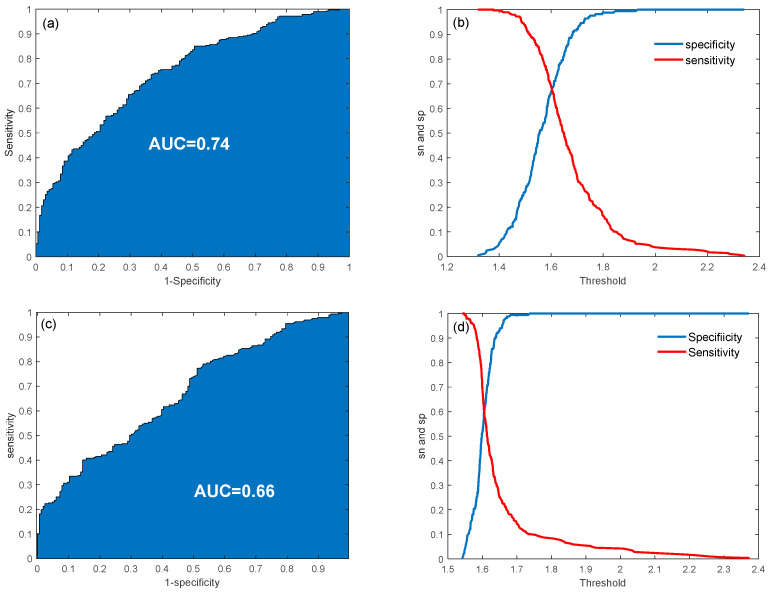
ROC curves and sensitivity/specificity plot for the classification problem using multiple variables (**a**,**b**) and PSA only (**c**,**d**).

From the validation procedure, the optimal penalization parameter for the LASSO model was determined to be 0.02. The model achieved an average classification accuracy of 0.67 ± 0.04 during cross-validation and 0.70 when applied to the entire dataset. Classification performance is depicted in Figure 2a,b, showing the receiver operating characteristic (ROC) curve along with sensitivity and specificity trends as the threshold varied. The area under the ROC curve (AUC) was 0.74, demonstrating an acceptable classification performance. These results highlight the effectiveness of LASSO regression in identifying critical biomarkers and improving classification accuracy within a biochemical framework. When using PSA alone, an accuracy of 0.63 and an AUC of 0.66 were obtained, meaning that the consideration of multiple variables in a multivariate model can improve the classification performance (Figure 2c,d).

### 3.5. Biplot Analysis

**Figure 3:**
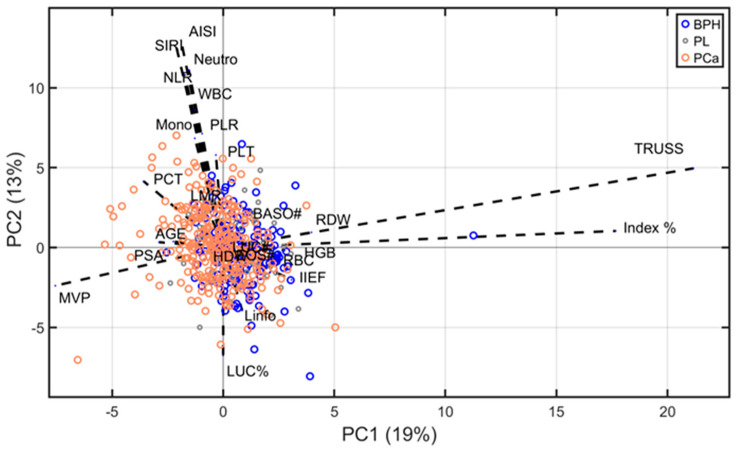
Biplot of PPCA showing the distribution of observations from three groups (BPH, PL, and PCa) along the first two principal components. Blue arrows indicate the loading values. For a clearer identification of variables’ contribution, loading values were multiplied 20 times.

The biplot presented in Figure 3 illustrates the first two principal components, PC1 (19% of variance) and PC2 (13% of variance), derived from the ABC-PPCA reconstruction. The plot reveals substantial overlap among the three clinical groups, underscoring the challenges of achieving clear classification between them. However, a partial separation is observed: several PCa observations predominantly exhibit negative values along PC1, while BPH observations tend to cluster more towards positive values, suggesting some degree of distinction.

The direction and length of the arrows in the biplot represent the contribution of each variable to the principal components, reflecting the variables’ loadings. Notably, TRUSS and Index % exhibit strong influences on PC1, correlating positively with the BPH cluster. Conversely, mean platelet volume (MPV), PSA, and age display stronger projections aligned with the PCa cluster. Variables such as HDW, eosinophils, and basophils contribute less significantly, as indicated by their shorter arrows near the origin.

Importantly, most of the findings obtained through PPCA analysis align with the results from prior analyses, reinforcing the robustness and consistency of the observed trends. These observations highlight the potential of specific variables to discriminate between clinical groups, even in the context of overlapping distributions.

### 3.6. Joint Analysis

**Figure 4:**
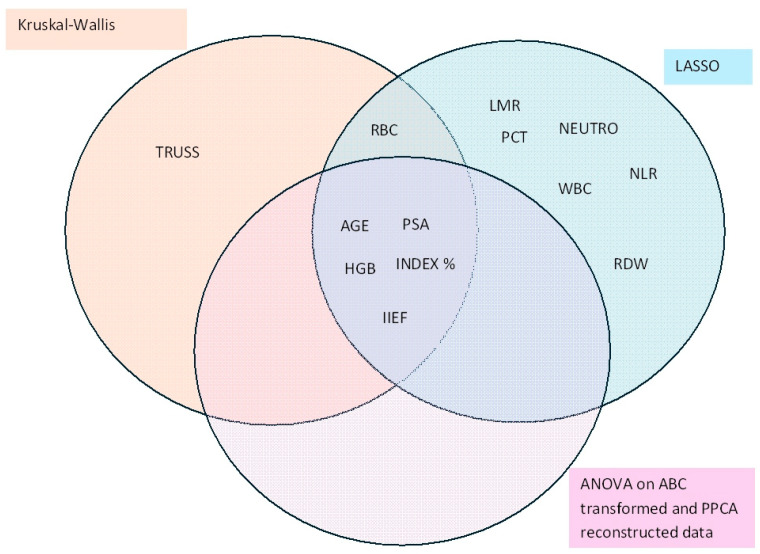
Euler–Venn diagram displaying the features for which the diagnosis effect resulted to be significant using three different methods.

To identify key variables, both univariate and multivariate methods were employed. The combined representation of results from these methods provides a robust assessment of the variables’ importance. The Euler–Venn diagram presented in Figure 4 illustrates the intersection of variables identified as significant by the Kruskal–Wallis test, ANOVA (both following FDR correction), and LASSO regression. Five variables, age, PSA, Index %, HGB, and IIEF, were consistently found to show significant changes across different diagnostic groups using all three methodologies.

As shown in the diagram, the LASSO regression was less conservative in variable selection compared to FDR-corrected *p*-values. This discrepancy is likely attributable to the stringent selection criteria imposed by the univariate methods, which inherently focus on reducing false positives. The intersection of results across multiple methodologies highlights the robustness of the selected variables and their potential utility in distinguishing between diagnostic groups.

## 4. Discussion

This study employed a combination of univariate and multivariate statistical techniques to identify biomarkers with strong potential for distinguishing PCa from BPH and PL. The integration of these complementary approaches strengthens the reliability of the findings and highlights the clinical utility of these biomarkers in improving diagnostic accuracy and patient stratification. The distribution of ISUP grades observed in our cohort, predominantly favoring lower-grade tumors (ISUP 1–2), reflects the typical heterogeneity of prostate cancer presentation at initial diagnosis. However, the significant proportion of patients with high-grade disease (ISUP 4–5) and the detection of metastatic disease in 7% of staged cases highlight the persistent clinical challenge of accurately stratifying risk at diagnosis. These findings underscore the importance of integrative biomarker models to more accurately distinguish indolent from aggressive forms of prostate cancer, thus optimizing treatment decisions and reducing overtreatment.

Several studies have confirmed that PSA is a widely used biomarker for prostate cancer (PCa) detection, although its specificity remains limited, leading to potential overdiagnosis and overtreatment [12,40]. Our study reinforces the role of PSA and identifies additional biomarkers such as age, Index %, and hemoglobin (HGB), which offer improved classification performance. The inclusion of inflammatory markers such as NLR and neutrophils aligns with emerging evidence supporting the role of systemic inflammation in PCa progression [41]. The observed associations between hematological parameters and PCa highlight the interplay between systemic inflammation and tumor development, as previously suggested by [21]. In contrast to previous studies that relied solely on univariate methods, our study employed a combined approach that incorporates multivariate techniques to refine biomarker selection. The adaptive Box–Cox transformation and probabilistic PCA used in this analysis effectively addressed data skewness and missingness, thereby enhancing the reliability of the results. The LASSO regression enabled the identification of the most relevant variables by integrating univariate results and mitigating potential data bias through advanced preprocessing techniques [42,43]. The classification accuracy of the model (70%) and the area under the curve (AUC = 0.74) indicate acceptable performance, supporting its potential for future clinical translation. These findings are consistent with current studies and clinical needs that emphasize the development of multimodal biomarker panels to improve specificity and reduce unnecessary biopsies [44]. By integrating these biomarkers into routine clinical practice, it may be possible to refine risk stratification models and reduce diagnostic uncertainty, while minimizing unnecessary interventions, in line with current recommendations for personalized medicine [45].

### 4.1. Biological Interpretation of Significant Biomarkers

Molecularly, elevated PSA reflects increased production by malignant prostate epithelial cells, often due to the disruption of tissue architecture and increased vascular permeability in cancerous lesions. A high Index % (percentage of free PSA) is typically associated with benign conditions, whereas a lower Index % supports malignancy, reflecting the higher proportion of complexed PSA in PCa. Reduced hemoglobin (HGB) levels may indicate cancer-associated anemia, which can result from chronic inflammation, bone marrow infiltration, or cytokine-mediated suppression of erythropoiesis. The IIEF score, reflecting sexual function, is often lower in PCa patients and may indirectly relate to tumor burden or hormonal changes affecting erectile physiology. These biomarkers together reflect systemic and local changes associated with PCa pathogenesis, offering a multidimensional diagnostic profile.

### 4.2. Strengths of the Study

This study employed a rigorous analytical framework combining univariate and multivariate approaches to identify and validate PCa biomarkers. The integration of statistical methods such as the ABC transformation, PPCA, and LASSO regression ensured robust data analysis, minimizing bias and improving reliability. A key strength of this study is the identification of biomarkers that are cost-effective and readily accessible in clinical settings. Age, PSA, Index %, HGB, and IIEF demonstrated consistent diagnostic value, reinforcing their relevance in differentiating PCa from BPH and PL. These findings align with previous reports suggesting that hematological and inflammatory markers could complement traditional PCa diagnostics [25]. Moreover, this study highlights the importance of multimodal biomarker integration, which has the potential to enhance diagnostic precision while reducing reliance on invasive procedures. The alignment of our findings with prior literature strengthens their potential applicability in clinical practice.

### 4.3. Limitations of the Study

Despite the strengths of this study, certain limitations must be acknowledged. While the cohort of 514 patients provides meaningful insights, larger multicenter studies are needed to validate the generalizability of these findings. The exclusion of the PL group from the multivariate analysis, due to its ambiguous separation, may have limited the ability to fully assess its diagnostic relevance. Future studies should incorporate larger PL cohorts to refine classification models. Another limitation is the absence of external validation. Although internal cross-validation confirmed the robustness of the model, future research should focus on validating these biomarkers in independent cohorts to confirm their clinical applicability. Additionally, this study employed a cross-sectional design, which provides a snapshot of biomarker performance but does not assess their prognostic value. Longitudinal studies will be necessary to evaluate how these markers change over time and their predictive power in disease progression. Moreover, this study recognizes that ethnic and geographic variability may significantly impact biomarker expression and diagnostic accuracy, underscoring the necessity for population-specific validation to enhance the broader applicability of our findings across diverse clinical settings. Addressing these limitations in future research will be crucial to strengthening the clinical utility of these biomarkers, ultimately refining PCa diagnosis and risk stratification. These findings contribute to the expanding body of evidence supporting the integration of advanced statistical methodologies in clinical biomarker discovery, with the potential to optimize patient outcomes and inform precision medicine strategies in prostate cancer management.

## 5. Conclusions

This study identified a robust panel of clinically accessible biomarkers—PSA, Index %, hemoglobin (HGB), age, neutrophils, NLR, and IIEF—with significant potential for stratifying patients with prostate cancer (PCa), benign prostatic hyperplasia (BPH), and precancerous lesions (PL). By integrating univariate and multivariate statistical methods, particularly LASSO regression and PPCA, we achieved improved classification performance (AUC = 0.74) compared to PSA alone. PSA, Index %, and age emerged as dominant contributors to diagnostic discrimination, while HGB and IIEF provided additional context related to systemic status and functional health. The inclusion of inflammatory markers such as NLR and neutrophil count supports the growing evidence of the role of systemic inflammation in PCa progression. These findings demonstrate that routinely collected hematological and inflammatory data can be effectively repurposed to support early diagnosis and risk stratification, thereby reducing unnecessary invasive procedures. Future validation in larger cohorts will be essential to confirm the clinical utility of this biomarker signature and its integration into personalized diagnostic workflows.

## Figures and Tables

**Table 1:** Kruskal–Wallis and ANOVA test results through *p*-values for various clinical parameters. ANOVA was conducted on PPCA reconstructed and ABC transformed data. Benjamini–Hochberg corrected *p*-values were corrected.

Kruskall–Wallis			ANOVA on ABC Transformed Data		
Variable	*p*-Value	Benjamini–Hochberg Corrected *p*-Value	*p*-Value	Benjamini–Hochberg Corrected *p*-Value	λ
AGE	<0.01	<0.01	<0.01	<0.01	2.87
PSA	<0.01	<0.01	<0.01	<0.01	0.56
Index %	<0.01	<0.01	<0.01	<0.01	0.47
WBC	0.12	0.21	0.07	0.15	0.25
RBC	0.01	0.04	0.04	0.09	−0.10
HGB	<0.01	0.003	<0.01	0.004	3.0
RDW	0.08	0.16	0.16	0.27	0.10
HDW	0.05	0.11	0.03	0.093	−1.33
MVP	0.70	0.83	0.63	0.81	1.11
PLT	0.57	0.75	0.65	0.81	0.06
PCT	0.67	0.83	0.81	0.86	0.16
NEUTROPHILS	0.03	0.08	0.02	0.07	0.03
LIMPHO	0.42	0.6	0.31	0.45	0.30
MONO	0.43	0.6	0.40	0.56	−0.50
EOS#	0.95	0.96	0.89	0.89	0.01
BASO#	0.85	0.93	0.83	0.86	0.01
LUC#	0.85	0.93	0.68	0.81	0.01
LUC%	0.38	0.6	0.19	0.29	0.46
LMR	0.04	0.10	0.14	0.25	−3.0
NLR	0.04	0.09	0.01	0.052	−0.3
PLR	0.89	0.93	0.72	0.82	−0.26
SIRI	0.02	0.07	0.01	0.05	−0.24
AISI	0.10	0.17	0.12	0.23	−0.32
IIEF	0.01	0.02	<0.01	<0.01	2.55
TRUSS	<0.01	<0.01	0.05	0.12	0.11

**Table 2:** Clinical, hematological, and inflammatory parameters in patients with BPH, PL, and PCa.

Variable	BPH (Mean ± SD)	PL (Mean ± SD)	PCa (Mean ± SD)	*p*-Value (ANOVA)	Regulation
Age (years)	65.0 ± 7.6	66.8 ± 8.5	69.6 ± 6.4	<0.001	PCa
PSA (ng/mL)	6.3 ± 3.8	8.6 ± 4.5	14.8 ± 13.1	<0.001	PCa
Hemoglobin (HGB) (g/dL)	14.5 ± 1.1	14.2 ± 1.2	13.7 ± 1.4	<0.001	BPH
White blood cells (WBC) (×10⁹/L)	6.5 ± 1.5	6.8 ± 1.7	7.0 ± 1.8	0.024	PCa
Neutrophils (×10⁹/L)	3.8 ± 1.2	4.1 ± 1.3	4.5 ± 1.5	<0.001	PCa
NLR	2.1 ± 0.9	2.4 ± 1.0	3.2 ± 1.4	<0.001	PCa
SIRI	1.1 ± 0.6	1.3 ± 0.7	1.7 ± 0.9	<0.001	PCa
Hemoglobin distribution width (HDW) (%)	2.4 ± 0.4	2.5 ± 0.5	2.7 ± 0.6	0.019	PCa
IIEF-5 Score	17.8 ± 5.3	16.5 ± 5.7	14.2 ± 6.0	<0.001	BPH

Data are expressed as mean ± standard deviation (SD). *p*-values were calculated using one-way ANOVA followed by post hoc Tukey’s test. Abbreviations: BPH, benign prostatic hyperplasia; PL, precancerous lesions; PCa, prostate cancer; PSA, prostate-specific antigen; HGB, hemoglobin; WBC, white blood cell count; NLR, neutrophil-to-lymphocyte ratio; SIRI, systemic inflammatory response index; HDW, hemoglobin distribution width; and IIEF-5, International Index of Erectile Function 5-item questionnaire.

**Table 3:** Post-hoc analysis for clinical parameters. The features that resulted to significantly change from ANOVA test are highlighted.

Variable	Class 0 vs. Class 1 (BPH vs. PL)	Class 0 vs. Class 2 (BPH vs. PCa)	Class 1 vs. Class 2 (PL vs. PCa)
Age	0.98	<0.01	<0.01
PSA	0.93	<0.01	<0.01
Index %	0.99	<0.01	<0.01
WBC	0.89	0.07	0.55
RBC	0.99	0.05	0.21
HGB	0.75	<0.01	0.01
RDW	0.93	0.26	0.32
HDW	0.31	0.36	0.03
MVP	0.99	0.63	0.87
PLT	0.76	0.69	0.98
PCT	0.82	0.89	0.95
NEUTROPHILS	0.87	0.02	0.40
LIMPHO	0.67	0.29	0.99
MONO	0.50	0.49	0.90
EOS#	0.88	0.97	0.94
BASO#	0.83	0.90	0.95
LUC#	0.68	0.83	0.87
LUC%	0.85	0.35	0.28
LMR	0.38	0.14	0.99
NLR	0.57	<0.01	0.62
PLR	0.93	0.697	0.98
SIRI	0.29	0.0104	0.92
AISI	0.59	0.0976	0.93
IIEF	0.99	<0.01	0.04
TRUSS	0.74	0.04	0.66

## Data Availability

The datasets generated and analyzed during this study are available from the corresponding author upon reasonable request. Researchers seeking access may be required to provide a brief proposal outlining the intended use and agree to confidentiality terms as per institutional policies.

## Supplementary Materials

The following supporting information can be downloaded at https://www.mdpi.com/article/10.3390/diagnostics15111385/s1: Section S1: Normality test; Section S2: Effect size analysis for the one-way ANOVA test; Section S3: Sensitivity analysis of data normality; and Section S4: Detailed Stratification of the Prostate Cancer Cohort According to ISUP Grade.

## Abbreviations

ABC	Adaptive Box–Cox transformation
AIOM	Italian Association of Medical Oncology
AISI	Acute inflammation systemic index
AUC	Area under the curve
BRCA1/BRCA2	Breast cancer gene 1/breast cancer gene 2
BPH	Benign prostatic hyperplasia
DRE	Digital rectal examination
FDR	False discovery rate
HGB	Hemoglobin
HDW	Hemoglobin distribution width
IIEF	International Index of Erectile Function
KW	Kruskal–Wallis
LASSO	Least absolute shrinkage and selection operator
MLR	Monocyte-to-lymphocyte ratio
mpMRI	Multiparametric magnetic resonance imaging
NLR	Neutrophil-to-lymphocyte ratio
PCa	Prostate cancer
PL	Precancerous lesions
PLR	Platelet-to-lymphocyte ratio
PPCA	Probabilistic principal component analysis
PSA	Prostate-specific antigen
RBC	Red blood cells
RDW	Red cell distribution width
ROC	Receiver operating characteristic
SIU	Italian Society of Urology
SIRI	Systemic inflammatory response index
SNV	Standard normal variate
TRUSS	Transrectal ultrasound scan
