# Supplementary material for: Diagnostic Stratification of Prostate Cancer Through Blood-Based Biochemical and Inflammatory Markers
Source: Diagnostics (Basel). 2025 May 30;15(11):1385. doi: 10.3390/diagnostics15111385 (PMC12155507; doi:10.3390/diagnostics15111385)
Supplement: Supplementary file 1 [file diagnostics-15-01385-s001.zip › diagnostics-3621972-supplementary.pdf]

Advanced Statistical Approaches for Identifying Clinical and Hematological Biomarkers in Prostate Cancer Stratification

Donatella Coradduzza [1*], Leonardo Sibono [2], Alessandro Tedde [3], Sonia Marra [3], Maria Rosaria De Miglio [5], Angelo Zinellu [1], Serenella Medici [6], Arduino A. Mangoni [7,8], Massimiliano Grosso [2], Massimo Madonia [3] and Ciriaco Carru [1,4]

[1] Department of Biomedical Sciences, University of Sassari, Sassari, Italy.
[2] Department of Mechanical, Chemical, and Materials Engineering, University of Cagliari, Cagliari, Italy.
[3] Department of Medicine, Surgery and Pharmacy, University of Sassari, Sassari, Italy; Unit of Urology, University Hospital of Sassari (A.O.U. SS), Sassari, Italy.
[4] Unit of Oncology, University Hospital of Sassari (A.O.U. SS), Sassari, Italy.
[5] Department of Medicine, Surgery and Pharmacy, University of Sassari, Sassari, Italy.
[6] Department of Chemical, Physical, Mathematical and Natural Sciences, University of Sassari, Sassari, Italy.
[7] Department of Clinical Pharmacology, Flinders Medical Centre, Southern Adelaide Local Health Network, Adelaide, Australia.
[8] Discipline of Clinical Pharmacology, College of Medicine and Public Health, Flinders University, Adelaide, Australia
* Correspondence: dcoradduzza@uniss.it

S.1 - Normality test

A normality test was performed for each variable associated with each specific class. A Lilliefors test was used to check whether the ABC-PPCA transformed variable adhered to a Gaussian behavior. Table S1 shows the improvement provided by the methodology presented in this work. As can be observed, the normality for most of the variables resulted in benefits from the transformation. For example, the p-value for HGB resulted in an increase from 0.03 to 0.5 and 0.001 to 0.07 for the first and second classes, respectively. Other variables, including EOS#, BASO#, and LUC#, did not show any p-value variation due to the quasi-discrete nature of such variables. Indeed, the experimental values for BASO# and LUC# were mainly 0.1, 0.2, and 0.3, making them less sensitive to any transformation.

Table S1: p-values obtained from Lilliefors normality test. Distributions before and after transformation were assessed.

	Before transformation			After transformation		
Variable	Class 1	Class 2	Class 3	Class 1	Class 2	Class 3
PSA	0.001	0.001	0.001	0.193	0.022	0.001
Index %	0.022	0.500	0.003	0.500	0.500	0.241
WBC	0.014	0.179	0.007	0.440	0.500	0.500
RBC	0.065	0.010	0.001	0.500	0.003	0.030
HGB	0.034	0.001	0.001	0.500	0.074	0.001
RDW	0.001	0.022	0.001	0.001	0.104	0.001
HDW	0.001	0.020	0.001	0.070	0.100	0.001
MVP	0.283	0.383	0.001	0.335	0.347	0.001
PLT	0.290	0.500	0.002	0.500	0.500	0.500
PCT	0.500	0.200	0.001	0.287	0.500	0.443
NEUTROPHILS	0.001	0.002	0.001	0.024	0.159	0.284

LIMPHO	0.001	0.059	0.001	0.011	0.500	0.093
MONO	0.001	0.001	0.001	0.001	0.002	0.001
EOS#	0.001	0.001	0.001	0.001	0.001	0.001
BASO#	0.001	0.001	0.001	0.001	0.001	0.001
LUC#	0.001	0.001	0.001	0.001	0.001	0.001
LUC%	0.006	0.001	0.001	0.076	0.005	0.018
LMR	0.001	0.001	0.001	0.001	0.001	0.001
NLR	0.001	0.001	0.001	0.052	0.137	0.481
PLR	0.001	0.197	0.001	0.500	0.500	0.500
SIRI	0.001	0.001	0.001	0.038	0.420	0.500
AISI	0.001	0.001	0.001	0.247	0.500	0.500
IIEF	0.001	0.007	0.001	0.002	0.030	0.001
TRUSS	0.001	0.019	0.001	0.159	0.136	0.500

S.2 – Effect size analysis for the one-way ANOVA test

Table S2: Effect sizes (Cohen's *f*) for various variables analyzed using one-way ANOVA. Variables with a medium effect size ($f \geq 0.25$) are highlighted in bold, while those with a small effect size ($0.05 \leq f < 0.25$) are shown in grey bold. The highest effect sizes were observed for PSA (f=0.289) and Index % (f=0.292), indicating a medium effect. Most other variables exhibited small effect sizes.

Biomarker	Cohen's *f*
**PSA**	**0.289**
**Index %**	**0.292**
**WBC**	**0.101**
**RBC**	**0.114**
**HGB**	**0.167**
**RDW**	**0.0846**
**HDW**	**0.115**
**MVP**	0.0425
**PLT**	0.0407
**PCT**	0.0282
**Neutrophils**	**0.1225**
**Lymphocytes**	**0.0679**
**Monocytes**	**0.0598**
**EOS#**	0.0215
**BASO #**	0.0274
**LUC #**	0.0389
**LUV %**	**0.0814**
**LMR**	**0.0884**
**NLR**	**0.130**
**PLR**	0.0359
**SIRI**	**0.129**
**AISI**	**0.0916**
**IIEF**	**0.172**
**TRUSS**	**0.108**

S.3 – Sensitivity analysis of data normality

Figure S1 presents a sensitivity analysis of data normality across different λ values for two variables: "PSA" (a and c) and "Index %" (b and d), by showing the p-values of the Lilliefors test for normality across the three groups and their product.

For the PSA variable, normality is rejected for nearly all the λ values, except for a narrow peak centered around λ=0.5, where a sharp increase in p-values is observed, particularly for the BPH and PL groups. However, the PCa group consistently remains non-normal. The combined p-values in panel (c) confirm this trend, with a sharp peak at λ=0.56 but values close to zero otherwise, indicating a strong deviation from normality.

Conversely, for the index %, normality is achieved for a broader range of λ, particularly for BPH and PL, suggesting a better adherence to normality conditions. The product of p-values in panel (d) reinforce this observation, showing a pronounced peak for λ =0.47

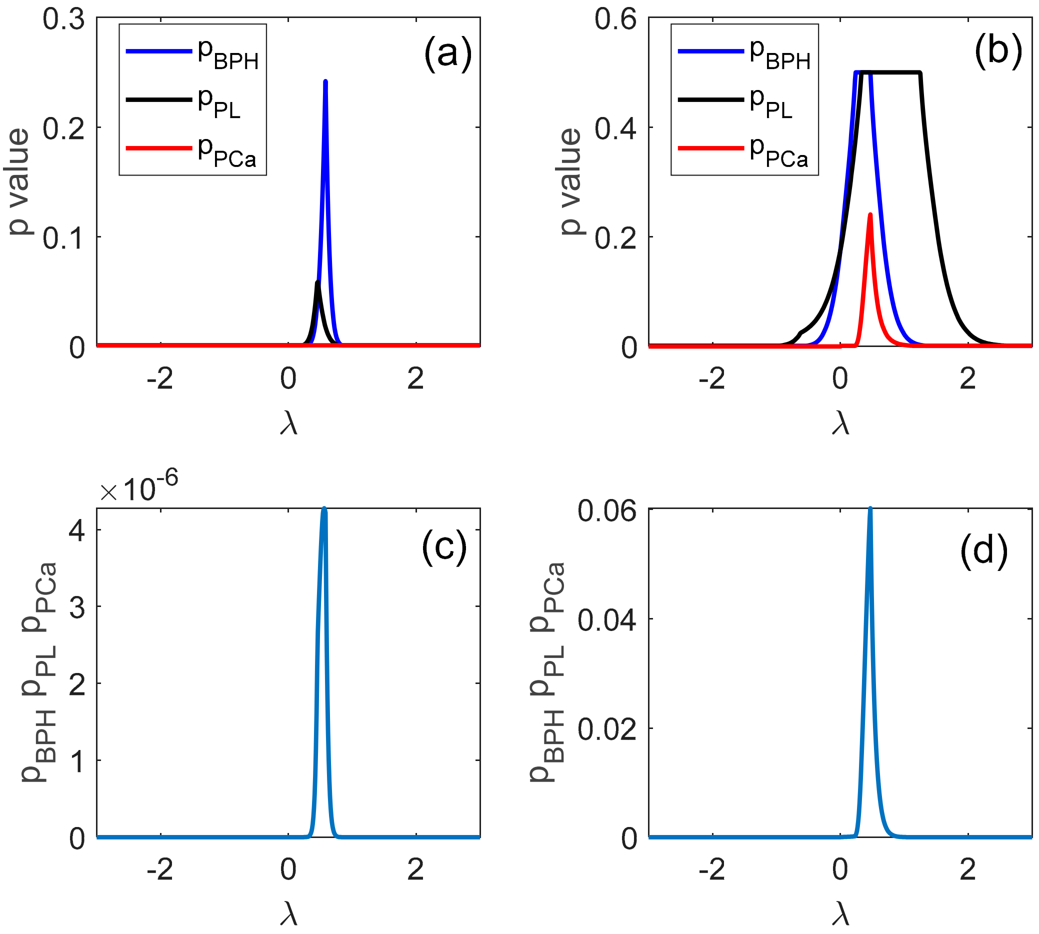

Figure S1. Sensitivity analysis of Lilliefors p-values across different λ values for PSA (a) and Index % (b). Respectively, the product of the three p-values displays the optimum λ identified by the algorithm (c,d).

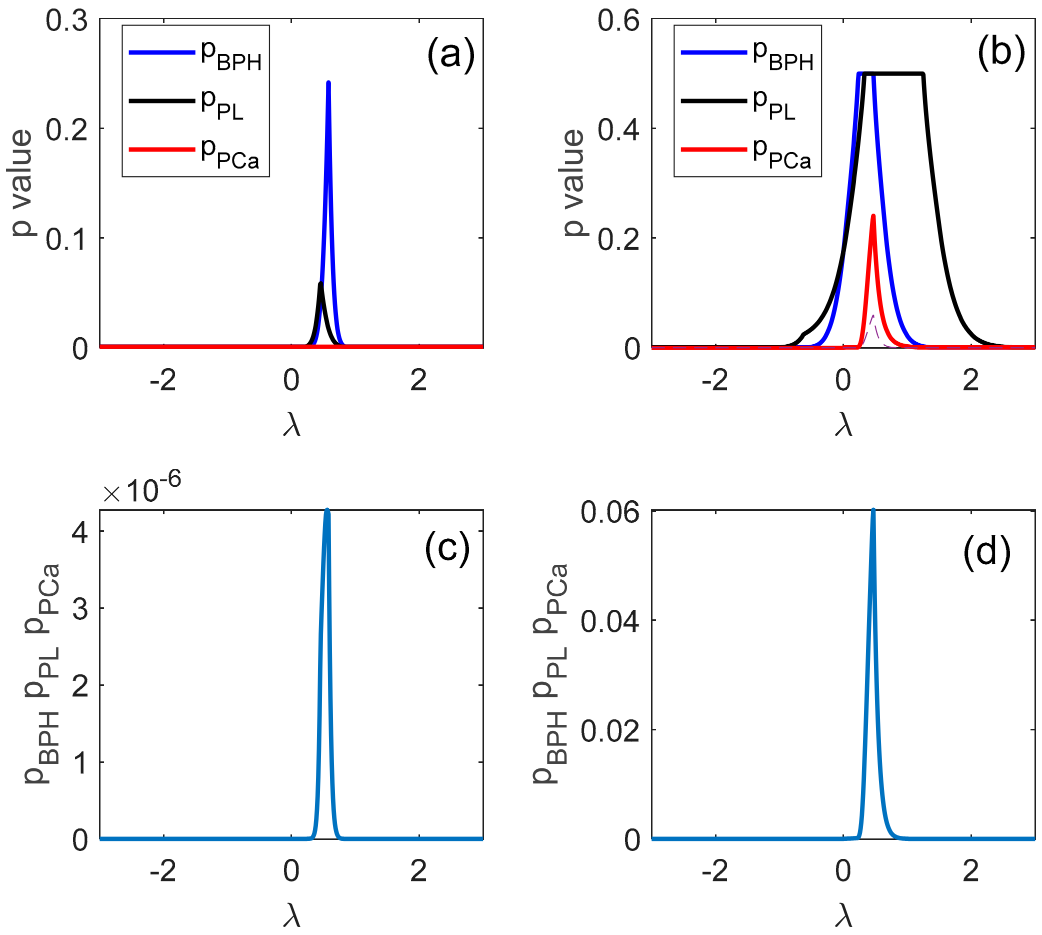

S.4 – Detailed Stratification of the Prostate Cancer Cohort According to ISUP Grade

To provide further stratification in the clinical interpretation of our findings, we performed a subgroup analysis of prostate cancer (PCa) patients according to the International Society of Urological Pathology (ISUP) grading system. This classification, which refines the Gleason scoring into five distinct prognostic categories, allowed us to assess variations in clinical and hematological parameters across different levels of tumor aggressiveness. The distribution of patients across ISUP grades was as follows: 42.2% of patients were classified as ISUP Grade 1, 13.1% as Grade 2, 12.4% as Grade 3, 20.0% as Grade 4, and 12.4% as Grade 5. This pattern reflects a predominance of lower-grade disease (Grades 1–2), while Grades 4 and 5 represent a smaller but clinically significant subset associated with more aggressive features.

The ISUP Grade Group Definition is as follows:

- Grade 1: Gleason ≤ 6 (3+3);
- Grade 2: Gleason 7 (3+4);
- Grade 3: Gleason 7 (4+3);
- Grade 4: Gleason 8 (4+4, 3+5, 5+3);
- Grade 5: Gleason 9–10 (4+5, 5+4, 5+5).

Table S3. Distribution of PCa Patients by ISUP Grade

ISUP Grade	Number of Patients	Percentage (%)
1	116	42.18
2	36	13.09
3	34	12.36
4	55	20.00
5	34	12.36
Total	275	100.00

There was a clear and progressive increase in PSA levels across ISUP grades, consistent with disease severity and tumor burden. Notably, TRUS levels tended to decrease in the highest grades, supporting their inverse association with tumor aggressiveness. Hemoglobin (HGB) concentrations showed a downward trend in higher ISUP groups, likely reflecting anemia of chronic disease, while WBC counts and inflammatory indices such as nNLR and SIRI were elevated in more advanced grades. These findings highlight the added value of integrating simple, routinely available clinical and inflammatory markers with histopathological grading systems and are shown in Table S4. This analysis further supports the prognostic utility of the ISUP classification and illustrates how conventional laboratory tests, such as complete blood count and PSA, can be meaningfully integrated into the risk stratification process.

Table 4. Descriptive Analysis by ISUP. Mann–Whitney Test Performed per Group Vs. BPH. *= p-value between 0.05-0.01; **= p-value between 0.01-0.001; and ***= p-value <0.001

VARIABLE	ISUP-1	ISUP-2	ISUP-3	ISUP-4	ISUP-5
AGE	71 (65-75)**	73 (65-78)*	75 (69-79)***	74 (70-79)***	75 (69-79)***
PSA	6 (4.7-8.7)	7.4 (4.4-11.7)*	14.1 (5.6-27.5)***	17.6 (6.9-46.1)***	15.4 (9.6-33.5)***
INDEX	14 (9-18)**	14 (8-21)**	13 (9.4-17.3)**	10 (7-12)***	11 (8.7-16)**
WBC	6.7 (5.6-8.4)	7.3 (6.1-8.5)**	7.9 (6.6-8.9)**	7.7 (6.3-9.2)**	6.7 (6.1-8.6)
RBC	5.2 (4.7-5.5)	5 (4.6-5.4)	4.9 (4.6-5.3)	5.1 (4.6-5.5)	4.9 (4.3-5.3)
HGB	14.2 (13.45-15)*	14.65 (13.45-15.15)	14.15 (13.6-15.6)	13.9 (12.93-15.07)*	13.9 (12.4-14.9)*
RDW	13.6 (13.2-16.67)	13.8 (13.37-15.25)	13.5 (12.878-14.45)	14 (13.57-15.62)	14.35 (13.4-15.8)
HDW	2.5 (2.4-2.8)	2.7 (2.3-3.1)	2.5 (2.4-2.8)	2.7 (2.5-2.9)	2.7 (2.5-2.9)*
MPV	8.3 (7.8-9.1)	8.3 (7.8-9.2)	8.5 (7.6-9.2)	8.7 (8-9.4)	8.5 (8.1-9.1)
PLT	213 (170.5-262)	231.5 (190.5-260.5)	240 (202-275)	202 (163.3-247.3)	234 (189-254)
PCT	1.78 (1.45-2.21)	1.93 (1.55-2.24)	2.02 (1.58-2.37)	1.77 (1.46-2.12)	1.91 (1.66-2.17)
NEUTRO	4.1 (3.1-5.3)	4.2 (3.5-5.3)*	4.6 (3.9-5.4)**	4.4 (3.6-5.6)**	4.1 (3.4-4.8)
LYMPHO	1.7 (1.3-2.3)*	2.2 (1.6-2.5)	2.1 (1.6-2.6)	2 (1.6-2.4)	1.9 (1.6-2.5)
MONO	0.4 (0.4-0.5)	0.5 (0.4-0.6)	0.5 (0.4-0.6)**	0.5 (0.4-0.6)	0.5 (0.4-0.6)
EOS	0.1 (0.1-0.2)	0.15 (0.1-0.2)	0.16 (0.1-0.3)	0.2 (0.1-0.3)	0.2 (0.1-0.3)
BASO	0 (0-0.1)	0 (0-0.1)	0.01 (0-0.1)	0 (0-0.1)	0 (0-0.1)

LUC#	0.1 (0.1-0.2)	0.1 (0.1-0.2)	0.1 (0.1-0.2)	0.1 (0.1-0.2)	0.1 (0.1-0.15)
LUC%	1.9 (1.5-2.5)	2.1 (1.47-2.65)	1.6 (1.3-2.2)*	1.8 (1.4-2.27)	1.75 (1.5-2.3)
LMR	0.31 (0.2-2.67)	0.3 (0.21-3.57)	0.4 (0.24-4.22)	0.25 (0.19-0.42)	0.26 (0.21-0.45)
NLR	2.39 (1.62-3.34)**	2.1 (1.63-2.81)	2.09 (1.61-2.62)	2.04 (1.71-3.14)	2.16 (1.71-2.59)
PLR	122.04 (90.68-164.25)	106.35 (90.23-147.13)	110.27 (91.25-137.5)	101.5 (76.4-136.41)	107.62 (78.89-150.63)
SIRI	0.99 (0.64-1.57)*	0.96 (0.77-1.44)	1.11 (0.78-1.45)**	0.92 (0.7-1.88)*	0.96 (0.72-1.55)
AISI	210.72 (124.75-409.41)	209.3 (149.55-318.99)	238.19 (165.11-453.75)**	198.06 (119.25-388.25)	212.3 (152.63-390.15)
IIEF	18 (10.25-23)	17 (9.25-22.25)*	15 (7-21)**	15 (7-18)**	17 (10.75-21)*
TRUSS	45 (34-60)***	48 (31.72-58.75)*	43 (37.25-49.75)**	46 (36-58)**	44 (32.9-54.25)**
